# miR-124 and miR-203 synergistically inactivate EMT pathway via coregulation of ZEB2 in clear cell renal cell carcinoma (ccRCC)

**DOI:** 10.1186/s12967-020-02242-x

**Published:** 2020-02-11

**Authors:** Jiajia Chen, Yuqing Zhong, Liangzhi Li

**Affiliations:** School of Chemistry, Biology and Materials Engineering, Suzhou University of Science and Technology, No. 1 Kerui Road, Suzhou, 215011 China

**Keywords:** MicroRNA synergism, Renal cell carcinoma, EMT pathway, Biomarker

## Abstract

**Background:**

Clear cell renal cell carcinoma (ccRCC) is one of the most aggressive urological malignancies. MicroRNAs (miRNAs) are post-transcriptional gene regulators in tumor pathophysiology. As miRNAs exert cooperative repressive effects on target genes, studying the miRNA synergism is important to elucidate the regulation mechanism of miRNAs.

**Methods:**

We first created a miRNA-mRNA association network based on sequence complementarity and co-expression patterns of miRNA-targets. The synergism between miRNAs was then defined based on their expressional coherence and the concordance between target genes. The miRNA and mRNA expression were detected in RCC cell lines (786-O) using quantitative RT-PCR. Potential miRNA-target interaction was identified by Dual-Luciferase Reporter assay. Cell proliferation and migration were assessed by CCK-8 and transwell assay.

**Results:**

A synergistic miRNA–miRNA interaction network of 28 miRNAs (52 miRNA pairs) with high coexpression level were constructed, among which miR-124 and miR-203 were identified as most tightly connected. ZEB2 expression is inversely correlated with miR-124 and miR-203 and verified as direct miRNA target. Cotransfection of miR-124 and miR-203 into 786-O cell lines effectively attenuated ZEB2 level and normalized renal cancer cell proliferation and migration. The inhibitory effects were abolished by ZEB2 knockdown. Furthermore, pathway analysis suggested that miR-124 and miR-203 participated in activation of epithelial-to-mesenchymal transition (EMT) pathway via regulation of ZEB2.

**Conclusions:**

Our findings provided insights into the role of miRNA–miRNA collaboration as well as a novel therapeutic approach in ccRCC.

## Background

Renal cell carcinoma (RCC) is the 3rd most prevalent urological malignancy with mortality at over 40% [1]. clear cell renal cell carcinoma (ccRCC) is the major subtype of RCC [2]. MicroRNAs (miRNAs) are master gene regulators that may silence genes at the post-transcriptional level via transcript degradation or translational repression. miRNAs are extensively implicated in fundamental biological processes and aberrant microRNA profiles have been reported in various cancers including RCC [3–5].

miRNA synergism is an important miRNA regulation mechanism, in which several miRNAs cooperate to regulate individual targets. Previous research has demonstrated that several miRNAs may function in a synergistic way to regulate their targets [6–9].

Tumorigenesis tends to be mediated by multiple miRNAs. For instance, the combination of miR-93 and miR-106a provide an accurate signature for prostate cancer diagnosis [10]. A panel of miR-31 and miR-146a play important roles in the activation of pancreatic stellate cells, which may contribute to pancreatic fibrosis [11]. In RCC, miR-215, miR-194 and miR-192 converge on the same target to suppress tumor development [12]. The synergism of miRNAs may optimize the regulatory efficacy of miRNAs at low abundance. Studying miRNA synergism could provide information on miRNA functions at systems level.

The main objective of this study is to reveal the functional association of the miRNAs in ccRCC carcinogenesis. We described a network-based procedure to identify tightly coregulating miRNAs, validated their expression, direct targets, function and evaluated their performance as binary classifiers in discriminating ccRCC from normal samples.

## Methods

### Data source

The matched miRNAs/mRNA expression profile of ccRCC GSE16441 [13] was downloaded from the Gene Expression Omnibus (GEO) repository. The data was derived from 34 specimens (17 RCC tumors and 17 corresponding non-tumor samples) based on platform GPL6480 and GPL8659.

### Differential expression analysis

LOWESS normalized average log fold changes were obtained. Limma package in R version 3.22.1 was utilized for differential expression analysis. P value was adjusted by Benjamin and Hochberg method for the significance analysis. |logFC| > 0.585 and FDR < 0.05 were used as the cut-off criteria.

### Constructing miRNA–miRNA co-regulating network

Pearson’s correlation coefficient was calculated between DE-miRNAs and DE-mRNAs, the cut-off was set as − 0.6. The candidate miRNA-mRNA pairs were filtered by in silico prediction in TargetScan [14] (PCT > 0.5) and the context + score wM,G was recorded for each pair (M, G).

**Fig. 1:**
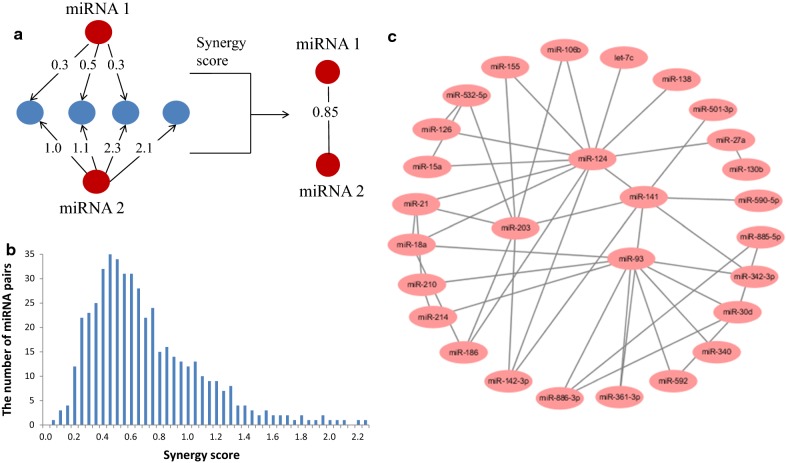
Synergism scores of ccRCC-specific miRNA co-regulation network. **a** The graphical presentation of the calculation process for synergism score; **b** Value distribution of miRNA synergism scores; **c** miRNA co-regulation network composed of candidate synergistic miRNA pairs (synergism score ≥ 1.400)

We quantified the degree of miRNA synergism by miRNA synergism score. Briefly, for each miRNA, an overall interaction score with all targets was calculated, ${\vec{\mathbf{V}}}_{M} \, = \left( {wM,G1,\,wM\,,G2\,, \ldots } \right)$, according to the context + score in TargetScan. The Pearson correlation of each ${\vec{\mathbf{v}}}_{M}$ vector is defined as the synergism score between each miRNA pair. Using this score as the weight of the link between two miRNAs, a miRNA–miRNA coregulation network was constructed. Fig.  1a is the graphical presentation of the calculation process with two miRNAs as an example.

### Functional synergism analysis of differentially expressed miRNAs

GO analysis was performed with the DAVID functional annotation tool. Pathway analysis was performed by MetaCore^TM^. The threshold was set as Benjamin-adjusted *p*-value < 0.05. For each miRNA pair, the number of the co-regulated GO terms was calculated.

### Cell culture and miRNA transfection

The human RCC cell line 786-O was obtained from the Gulou Hospital (Nanjing, China) and propagated at 37 °C with 5% CO_2_. Before transfection, cells were seeded to 6-well plates in a final density of 0.8 × 10^5^ cells per well and cultured in fresh medium without antibiotics. The miR-124 and miR-203 mimic, and controls were synthesized by GenePharma Biotechnology (Shanghai, China). 786-O cells were transfected with miR-124 mimic, miR-203 mimic, a mixture of both, or negative control precursor miRNA. 5 μg/ml Blasticidin was used for antibiotic selection.

### RNA isolation and qRT-PCR

Total RNA was isolated with the miRNeasy Mini Kit (Qiagen, Germany). For miRNA quantification, TaqMan miRNA assays was performed and U6 used as an internal control. PCR parameters were set as: denaturation at 92 °C for 5 min, amplification at 15 °C for 30 s and 72 °C for 45 s for 30 cycles. For mRNA quantification, PCR was done using gene-specific probes (Shenggong, Shanghai, China) and β-actin was used as an internal control. The relative miRNA/mRNA expression change was calculated with the 2^− △△Ct^ method.

### Luciferase reporter assay

The 3′-UTRs of ZEB2 containing the putative miR-124 or miR-203 binding site were amplified via PCR. Wild type (MT) 3′-UTR was cloned downstream of the firefly luciferase gene of the pmirGLO vector to construct pmirGLO-ZEB2-WT-3′UTR plasmid. The plasmid was mutated (MT) at the binding site for miR-124 or miR-203, to generate pmirGLO-ZEB2-MT-3′UTR. All constructs were verified via sequencing. The 786-O cell lines were inoculated in 24-well plates and cotransfected with miR-124 mimics, miR-203 mimics or scrambled mimics and WT/MT 3′UTR plasmids. Luciferase activity was measured 48 h after transfection.

### Cell migration assay

Cell migration assay was performed with the transwell system (24-well insert, pore size 8 μm; Millipore, USA). After 48 h transfection with the miRNA mimics, ZEB2-siRNA or negative control (NC), aliquots of 786-O cells were resuspended in FBS-free RPMI-1640 medium and plated into the upper chamber of the non-coated membrane. The lower chamber was filled with 30% FBS containing RPMI. After incubation for 24 h at 37 °C, cells were fixed with 4% formaldehyde and stained with 0.5% crystal violet for 5 min. The migrated cells were photographed (200 ×) and scored in 10 random fields per well under light microscope. Each test was performed in triplicate.

## Results

### Identification and evaluation of synergistic miRNA pairs

We first performed differential expression analyses for matched microRNA and mRNA profiles and constructed an initial miRNA–mRNA network according to miRNA–mRNA negative correlations and target information predicted for sequence complementation. As a result, we obtained a miRNA–mRNA network consisted of 28 miRNAs and 516 target genes.

Next, we calculated the synergism score of two miRNAs to identify synergistic miRNA pairs for further validation. For each miRNA, we used a vector ${\vec{\mathbf{V}}}_{M} \, = \,\left( {wM,\,G1,\,wM,\,G2, \ldots } \right)$ to represent its overall silencing effect on all the target genes. The *i*-th component, *wM, Gi*, is the silencing effect of microRNA *M* and the *i*-th target gene, based on the context + score in TargetScan.

The synergism score between miRNAs is defined as the Pearson correlation of their ${\vec{\mathbf{V}}}_{M}$ vectors, which represents the similarity of their regulation patterns. Based on the synergism score, DE-miRNAs co-regulating the same target gene were identified, based on which a DEmiRNA co-regulation network was constructed and visualized through Cytoscape software [15]. As a result, a miRNA–miRNA network consisted of 28 miRNAs and 58 regulations was constructed, in which a node represents a miRNA and an edge represents a ccRCC-specific cooperation between two miRNAs, and the synergism score was used as the weights of edges connecting the nodes. We measured the statistical significance of the synergism score by using the exact randomization tests.

Figure 1b illustrates the distribution of synergism score values for 406 random miRNA pairs. The distribution of miRNA synergism scores is asymmetric. The values of a majority (91.1%) of miRNA pairs are distributed between 0.2 and 1.3. Only 30 miRNAs pairs (7.4%) are distributed in the high score region. As expected, we failed to observe extensive miRNA synergism in the whole miRNA regulation network and only a small proportion of miRNA pairs were found to show adequate synergism. The highest synergism score 2.31 was obtained by miR-124 and miR-203 which strongly implied a potential coregulation of these two miRNAs.

### Dissection of Gene Ontology (GO) highlights microRNA synergism

To determine functional coordination of miRNA coregulatory pairs, we performed GO functional analysis and the significantly enriched GO terms were filtered (*p *< 0.05). The number of co-regulated GO terms N for each miRNA pair was calculated and used as an indicator of functional similarity. According to the number of co-regulated GO terms, the miRNA pairs can be divided into two groups, low synergism group (n ≤ 10) and high synergism group (n > 10).

As a control for significance, we randomly selected a group of 28 miRNAs and calculated pairwise the number of co-regulated GO terms. Compared with the control group, we noted that more of the identified pairs (370 out of 406) fell within the high coregulation group compared with 60% in random sets, suggesting that these pairs have higher functional similarity than random pairs and that functional synergistic relationships exist between the corresponding DEmiRNAs.

### miR-124 and miR-203 expression were inversely related with ZEB2

**Fig. 2:**
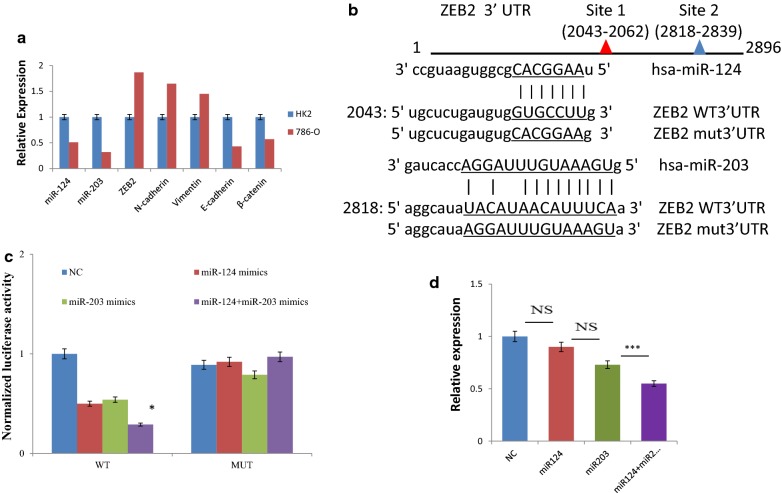
ZEB2 is directly targeted by miR-124 and miR-203. **a** Relative expression levels of ZEB2, miR-124 and miR-203 expression levels in renal cancer cell line (786-O) and normal immortalized renal cell line (HK2) as assessed by qRT-PCR; **b** schematic representation of the seed sequence of miR-124 and miR-203 and their putative binding sites in ZEB2 3′-UTR; **c** result of Luciferase assay in 786-O cell lines cotransfected with the ZEB2 wild type 3′UTR or ZEB2 mutated 3′UTR reporter plasmids and either miR-124/miR-203 mimics or scrambled miRNA mimics. The result is given in relation to the control luciferase activity. Data are calculated as mean ± SD of triplicates. **d** Effect of miRNA restoration on ZEB2 expression determined by qRT-PCR. miR-124 and/or miR-203 were transfected in 786-O cell lines (*p < 0.05; **p < 0.01; *ns* not significant)

qRT-PCR was performed to confirm an inverse relationship between ZEB2 and miR-124/miR-203 expression. The relative level of ZEB2 expression in RCC cell line 786-O versus HK-2 is shown in Fig. 2a. The data demonstrated that consistent with results in microarrays, the expression of miR-124 and miR-203 was attenuated and ZEB2, a transcriptional repressor, was up-regulated in 786-O cell lines.

### ZEB2 is a direct target of miR-124 and miR-203

Because of the cooperation between miR-124 and miR-203 in the regulation of ZEB2, they were selected for further investigations. The putative miR-124 or miR-203 binding sites in ZEB2 mRNA are illustrated in Fig. 2b. To ascertain ZEB2 as a direct target of miR-124 and miR-203, we created plasmids encoding the WT or MT 3′UTR regions of ZEB2 mRNA, which were co-transfected together with miR-124 or miR-203 mimics or scrambled mimics into 786-O cells.

As illustrated in Fig. 2c, a consistent reduction of luciferase activity upon either miR-124/miR-203 transfection suggested that both miRNAs repress ZEB2 directly. The luciferase activity was reduced with wild type luciferase construct by 50% and 46% after miR-124 and miR-203 overexpression, respectively. Combination of both miR-124 and miR-203 led to a decrease of 71%. There was no significant decrease in luciferase activities with the mutated luciferase construct.

### Effect of miR-124 and miR-124 overexpression on ZEB2 expression

To evaluate the role of miR-124 and miR-203 on the ZEB2 expression in ccRCC, we transfected single or both miRNAs mimics into 786-O cell lines to restore the expression of miRNA. Here, the ZEB2 expression was attenuated by 11% and 27% after single transfection of miR124 and miR203 respectively. The joint overexpression of both miR-124 and miR-203 provided an additional decrease in ZEB2 expression of 45% (Fig. 2d).

### miR-124 and miR-203 negatively regulate cell proliferation and migration

**Fig. 3:**
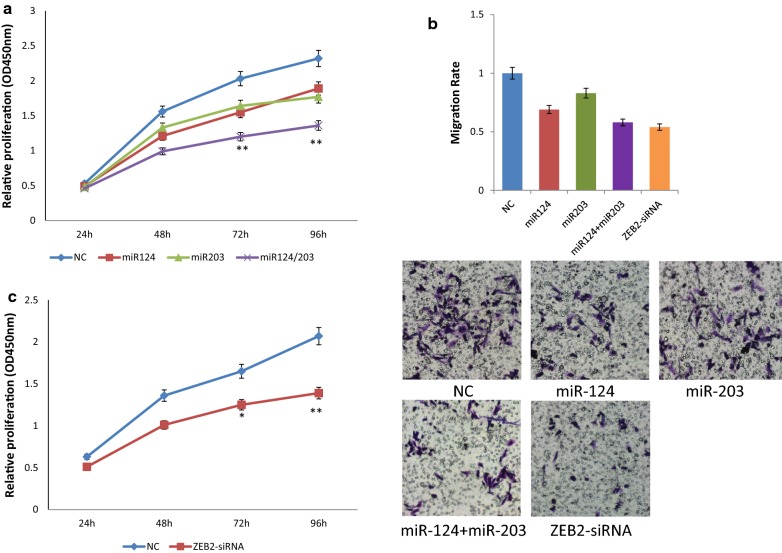
miR-124 and miR-203 suppressed tumorigenicity in vitro via ZEB2. **a** Cell proliferation analysis by CCK-8 assay upon miR-neg, miR-124, miR-203 and miR-124/203 transfection; **b** migration assay in 786-O cell line upon miR-neg, miR-124, miR-203, miR-124/203 and ZEB2-siRNA transfection. Migration rate is given as the ratio to negative control (mean ± SEM); **c** cell proliferation analysis by CCK-8 assay after transfection by miR-neg and ZEB2-siRNA

To analyze the effect of miR-124 and miR-203 on cell proliferation and migration, we transfected 786-O cell lines with miR-neg, miR-124, miR-203, or miR-124/203 scramble, followed by functional assays. CCK-8 assay indicated that both miRNAs had a strong inhibitory effect on the proliferation of 786-O cells compared with negative control (Fig. 3a). Transwell migration assay showed that miR-124 was efficient in inhibiting migration and miR-203 also seemed to inhibit migration, although not as efficient as miR-124 (Fig. 3b). Moreover, the combination of two miRNAs tended to have an enhanced inhibitory effect on proliferation and migration compared with the single miRNAs.

### ZEB2 knockdown phenocopies miR-124/miR-203 overexpression

To further investigate the mechanism underlying the tumor suppressive effect of miR-124/miR-203, we knockdowned ZEB2 in 786-O cells by transfecting ZEB2-siRNA or negative control, followed by functional analysis. As a result, the proliferation of ZEB2-silenced 786-O cells significantly decreased compared with negative control, similar to the phenotype after miR-124/203 transfection (Fig. 3c). The migration rate of ZEB2-silenced 786-O cells also decreased (Fig. 3b), supporting the idea that miR-124/203 repressed cell proliferation and migration via regulation of ZEB2 in ccRCC.

### miR-124 and miR-203 regulate renal cancer migration and proliferation via EMT

**Fig. 4:**
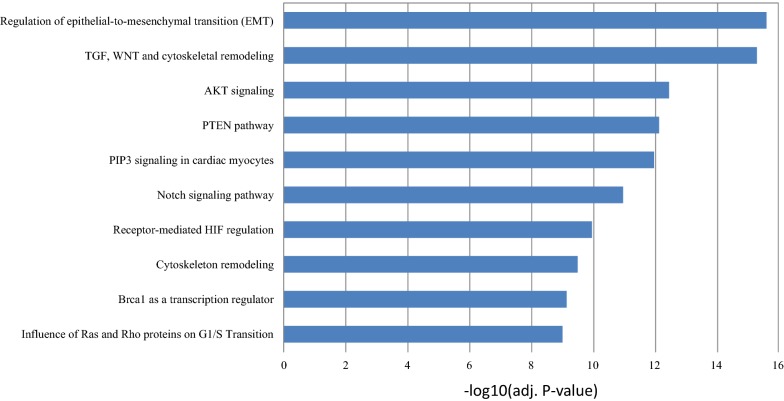
The top 10 significant pathways enriched with miRNA targets. The statistical significance was negative 10-based log transformed adj. *p* value

We have confirmed that miR-124 and miR-203 could repress ZEB2, a transcriptional repressor of key regulators of epithelial differentiation and an activator of EMT. We further performed pathway analysis to rank the pathways that are significantly enriched with target genes of miR-124 and miR-203. The top 10 enriched pathways are listed in Fig. 4. The result validated that the target genes of miR-124 and miR-203 were most significantly enriched in EMT regulation pathway. Other enriched pathways e.g. TGF, WNT and cytoskeletal remodeling, AKT signaling, PTEN pathway, PIP3 signaling in cardiac myocytes, Notch signaling pathway and receptor-mediated HIF regulation are also widely reported to have regulatory roles on EMT.

EMT has been widely reported to implicate in tumor migration and metastasis. EMT is characterized by a wide range of changes of marker molecules, e.g. upregulation of N-cadherin and vimentin, and redistribution of β-catenin from E-cadherin. Both in RCC tissues and RCC cell lines where miR-124 and miR-203 were downregulated, we observed reduced expression of E-cadherin and β-catenin (epithelial marker) as well as induced level of N-catenin and vimentin (mesenchymal markers), indicating that loss of both miRNAs significantly increased the migration and proliferation of ccRCC cells via regulating the key elements in EMT.

## Discussion

Previous studies have confirmed that a large number of miRNAs was involved in tumorigenesis. The role of miRNA cooperation in cancer progression has also attracted wide research interest. In this paper, we established a synergistic miRNA networks by calculating synergism score that was based on both transcriptomic data and miRNA-target interaction. miRNA synergism score which quantitatively evaluated the degree of regulation similarity between two miRNAs, can give highly probable co-regulating miRNAs.

As a result, a highest synergism score of 2.31 was obtained for the miRNA pair miR-124 and miR-203, which placed this pair in the center of the miRNA coregulation network. Both miR-124 and miR-203 are known to be aberrantly expressed and play tumor suppressive roles in a wide range of human cancers. Deregulation of miR-124 has been demonstrated in liver cancer [16], breast cancer [17–19], colorectal cancer [20], lung carcinoma [21] and nasopharyngeal carcinoma [21]. miR-203 has also been shown to be deregulated in multiple types of tumor entities, e.g. melanoma [22, 23], ovarian cancer [24], lung adenocarcinoma [25], myeloma pancreatic cancer [26, 27], breast cancer [28], glioblastoma [29], neuroblastoma [30].

Recent evidence pointed out a specific tumor-suppressive or oncogenic effect of miR-124 and miR-203, that both miRNAs can mediate tumorigenic processes, e.g. proliferation, migration, apoptosis and metastasis [31].

Butz et al. [32] pinpointed miR-124 as a key miRNA contributing to ccRCC aggressiveness by targeting CAV1 and FLOT1 using a miRNA-target network. Methylation of miR-124 is more frequent in malignant ccRCC than in normal kidney tissues [33]. Hypermethylation of miR-124 is strongly associated with advanced RCC stage, differentiation grade and an increased risk of recurrence [34]. The downregulation of miR-124 may serve as a predictor of survival [32]. By sponging miR-124, HOTAIR as ceRNA unregulated ST8SIA4 and promoted the proliferation and metastasis in RCC [35, 36].

miR-203 acts as a suppressor in many cancer types including ccRCC. miR-203 is frequently downregulated in RCC cell lines and specimens and the reduced expression of miR-203 in ccRCC primary tumors correlates with poor prognosis and metastasis. While overexpression of miR-203 inhibited cell proliferation, migration, invasion, and induced apoptosis and cell cycle arrest [37–39]. Xu et al. found that miR-203 leads to tumor suppression by targeting FGF2 [37] and exerts anti-metastatic activity via inhibition of EMT and metastatic genes [39]. lncRNAs e.g. SNHG14 [38] and HOTAIR [39] sponge miR-203 as ceRNA and promote ccRCC migration and invasion.

Interestingly, miR-203 was also reported to act as an oncogene in RCC and an indicator of poor prognosis [40]. Therefore, miR-203 may have different roles at different stages of ccRCC, either as a tumor suppressor or an oncogene.

Although the role of miR-124 and miR-203 in the pathogenesis of renal cancer has been established, this is the first report that implicates the collaboration of miR-124 and miR-203 in the pathogenesis of ccRCC.

In this research we have demonstrated that miR-124 and miR-203 were downregulated in cell lines 786-O in contrast to non-tumorigenic renal tubular epithelial HK-2 cells. Intriguingly, we revealed a novel cooperative regulation of miR-124 and miR-203. Gene Ontology enrichment revealed that they jointly participated in the same functional processes, which suggested potential miRNA synergism. miR-124 and miR-203 tended to inhibit synergistically specific oncogenic target ZEB2 and further cell proliferation and migration in RCC cell lines.

We found a negative correlation between ZEB2 and miR-124/miR-203 levels in both ccRCC tissue and cell lines. Dual-luciferase reporter assay showed that after transfection of miR-124 and miR-203, either jointly or separately, the luciferase activity in the ZEB2 wild-type reporter gene decreased but no change was detected for the mutant type, which confirmed that ZEB2 was directly targeted by miR-124 and miR-203. A significant decrease was observed for ZEB2 level after transfection of each miRNA. Notably, by simultaneous overexpression of miR-124 and miR-203, we observed a higher decrease in ZEB2 expression, which convinced the synergistic effect of miR-124 and miR-203.

As previously shown and confirmed by our data, miR-124 and miR-203 transfection can also inhibit proliferation of renal cancer cells. Additionally, in 786-O cell lines, both miRNAs exhibit inhibitory effects on cell migration. Co-transfection led to more effective inhibition of migration behavior than the single transfections, which could be attributed to the synergism between the miRNAs.

As demonstrated by miR-124 and miR-203, the repression effects of single miRNA on target gene is limited. However, miRNA pair exerts reinforced inhibitory effect on shared target genes and hence for ZEB2 we obtained a significantly enhanced inhibitory effect in the case of co-transfection of both miRNAs. Therefore, co-transfection of miRNA-mimics could provide an efficient strategy for miRNA-restoration based cancer therapy. This is possible, if the seed sequences of multiple miRNAs are complementary to the same mRNA 3′UTR region.

In addition, we have validated that ZEB2 is an important target. We silenced ZEB2 in 786-O cells and found that the proliferation and migration were attenuated, similar to the phenotype observed after miRNA-overexpression in 786-O cells, implying that the tumor suppressive role of miR-124 and miR-203 may be mediated mainly through targeting ZEB2.

ZEB2 is a two-handed zinc finger transcription repressor which was found to be overexpressed in several cancer cell lines. It represses important mediators of epithelial differentiation [41] and induces EMT [42]. EMT was first discovered in embryonic development. After the adult epidermal cells were damaged, the corresponding EMT phenomenon also appeared. However, in the process of tumor cell development, EMT will also cause the tumor cells to lose the properties of some epithelial cells to acquire the properties of some mesenchymal cells, and also enable the tumor cells to obtain stronger invasion and detachment ability. EMT is reactivated during tumorigenesis and has been related to tumor migration, invasion and metastasis [41]. Moreover, cancer cells can utilize EMT to acquire cancer stem cell characteristics through the modulation of miRNAs [43, 44].

The mechanism of action of EMT varies in different tissues, under different malignant conditions of tumor cells, and in different intracellular and extracellular pathways. During the development of EMT, epithelial cells are lost in polarity. Contact with surrounding cells and stromal cells is reduced, intercellular interactions are reduced, and cell migration and exercise capacity are enhanced. At the same time, the phenotype of cells changes, and the epithelial phenotype is lost. For example, keratin filaments, E-cadherin, and decreased levels of E-cadherin can lead to decreased adhesion of cells, which makes cells susceptible to invasion and metastasis, and expression of E-cadherin loss has been considered the most prominent feature of EMT. At the same time, the cells obtained an interstitial phenotype, such as Vimentin, N-cadherin and other expression increased.

**Fig. 5:**
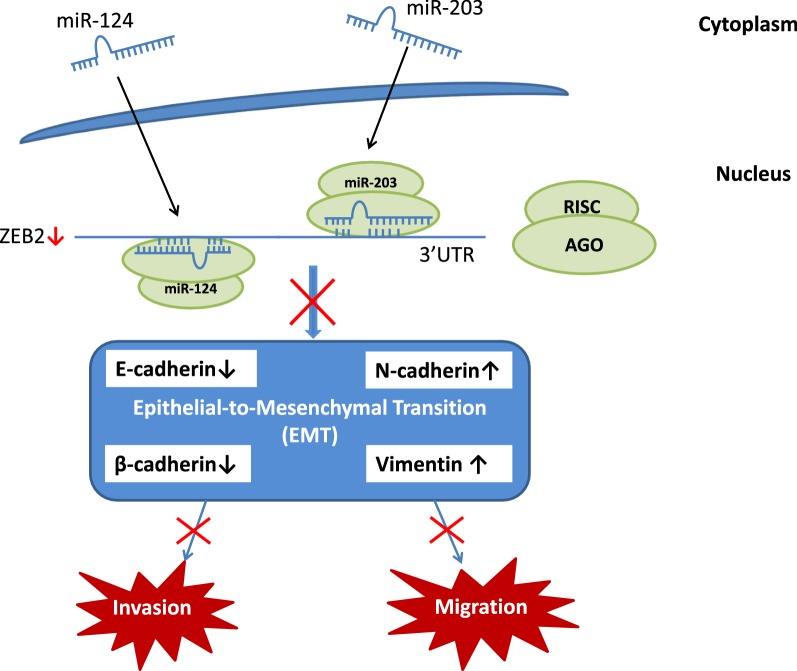
Schematic diagram of miRNA-mediated EMT inactivation in ccRCC

In line with previous research, in RCC cell lines we observed down-regulation of E-cadherin and β-catenin, as well as up-regulation of N-cadherin and Vimentin, as illustrated in Fig. 2a. It is likely that up-regulation of miR-124 and miR-203 in ccRCCs might be involved in the inactivation of EMT pathway via down-regulation of ZEB2. A schematic diagram of miRNA-mediated EMT inactivation in ccRCC is outlined in Fig. 5.

The pathway analysis revealed that the target genes of miR-124 and miR-203 were significantly enriched in EMT regulation pathway. Other top enriched pathways are also associated with EMT.

For example, in TGF, WNT and cytoskeletal remodeling pathway, TGF is considered to be the most critical factor in inducing EMT during developmental processes, carcinogenesis, and other pathological conditions. In some in vitro cultured epithelial cell lines, simple TGF-β stimulation could induce EMT. The TGF-β signaling-mediated EMT can be achieved by the classical Smad pathway or by the non-Smad pathway. In the classical smad pathway, TGF-β signaling activates Smad2 and Smad3 via a tetrameric complex type I and type II receptor (TbRI and TbRII) and binds to Smad4. The Smad complex will transfer to the nucleus together with transcription factors to mediate inhibition or activation of the target gene. At the same time, Smad complex can also induce miRNA expression in the nucleus, thereby inhibiting the expression of marker proteins in epithelial cells and promoting protein expression of mesenchymal cells, thereby promoting EMT. In the TGF-β signaling-mediated non-Smad signaling pathway, it activates the PI3K-AKT-mTOR signaling for transcriptional regulation, and activated AKT can also trigger transcriptional regulation by inhibiting ribonucleoprotein E1 (hnrnpe1). TGF-β also affects the activity of several other EMT trigger signaling pathways, such as Notch, Wnt, and integrin signaling pathways. On the other hand, TGF-β will regulate Rho GTPase to ubiquitinate and degrade by regulating changes in the cytoskeleton, thereby reducing the tight junctions between cells.

Wnt, another member of the TGF, WNT and cytoskeletal remodeling pathway, is also closely related to the occurrence and development of many human tumors. Overexpression of Wnt is found in multiple cancer entities. When Wnt binds to its receptor frizzled protein (Frz), Frz acts on the intracytoplasmic protein, which inhibits the activity of GSK-3β, blocks the degradation pathway of β-catenin, and makes β-catenin in the cytoplasm. It accumulates and enters the nucleus, interacting with T-cell factor (TCF/LEF) to activate transcription of downstream target genes, e.g. c-myc, cyclinD1, WISP, etc. Among them, c-myc can induce the morphological changes of mammalian epithelial cells and enhance the non-anchor-dependent growth ability of cells; while WISP has a certain relationship with the occurrence of colon cancer. Therefore, activation of these genes can promote the development of EMT and initiate tumor growth and metastasis procedures.

In this study, we proposed an in silico methodological pipeline for miRNA–miRNA network construction and identification, followed by in vitro functional analysis. However, the possible synergism between miR-124 and miR-203 and their function were only validated in the molecular and cellular level. It’s true that in vivo experiments are closer to physiological conditions, more scientific and representative than in vitro approaches. A preclinical proof-of-concept experiment using in vivo methods is very important for result explanation and carcinogenic analysis. In vivo animal and clinical research are required to further justify our findings in the future.

## Conclusion

In conclusion, by identifying the cooperative effect of miR-124 and miR-203 in the regulation of ZEB2, we have provided a deeper insight into the role and scale of miRNA–miRNA collaboration in ccRCC. The novel cooperative effect of miRNAs in tumor suppression might be promising as a novel therapeutic approach for ccRCC.

## Data Availability

The datasets analyzed during the current study are available in the NCBI GEO repository, GSE16441 in https://www.ncbi.nlm.nih.gov/geo/query/acc.cgi?acc=GSE16441. The data generated during this study are available from the corresponding author upon reasonable request.

## Abbreviations

ccRCC: Clear cell renal cell carcinoma
LIMMA: Linear models for microarray data analysis
ZEB2: Zinc finger E-box-binding homeobox 2
EMT: Epithelial-to-mesenchymal transition
RCC: Renal cell carcinoma
GEO: Gene Expression Omnibus
LOWESS: Locally weighted scatterplot smoothing
DE: Differentially expressed
DAVID: The Database for Annotation, Visualization and Integrated Discovery
GO: Gene ontology
AUC: Area under the curve
